# ST-Strecken-Hebungen und Lungenödem – „nur“ ein ischämiebedingter kardiogener Schock?

**DOI:** 10.1007/s00063-025-01258-9

**Published:** 2025-03-27

**Authors:** Maximilian Eichner

**Affiliations:** https://ror.org/03b0k9c14 grid.419801.5 0000 0000 9312 0220 4. Medizinische Klinik/Zentrale Notaufnahme, Universitätsklinikum Augsburg, Stenglinstraße 2, 86156 Augsburg, Deutschland

**Keywords:** Neurogenes Lungenödem, Takotsubo-Kardiomyopathie, Intrazerebrale Blutung, Basilarisaneurysma, ST-Strecken-Hebungen

## Falldarstellung

Eine 54-jährige Patientin wurde luftgebunden nach präklinischer Intubation unter der Voranmeldung „V. a. Aortendissektion, DD Myokardinfarkt, DD intrazerebrales Geschehen“ in unsere Notaufnahme transportiert.

Die Patientin hatte ursprünglich von zu Hause aus selbst den Notruf verständigt und über die plötzliche Unfähigkeit, ihre Extremitäten bewegen zu können, geklagt. Der Rettungsdienst und die Notärztin fanden nach Wohnungsöffnung eine tief bewusstlose, im Flur liegende Frau mit einem Wert auf der Glasgow Coma Scale (GCS) von 3 und schaumigem Sekret um den Mund vor. Der Blutdruck lag bei 210/150 mmHg systolisch, die Herzfrequenz betrug 140/min bei Sinusrhythmus am Monitor, und die periphere Sauerstoffsättigung lag bei 54 %. Die Pupillen waren isokor, mittelweit und nicht lichtreagibel. Eine dezidierte neurologische Untersuchung fand am Einsatzort nicht statt, da bei schwerer Bewusstseinsstörung mit respiratorischer Insuffizienz die sofortige Intubation erfolgte. Die Narkoseeinleitung erfolgte mit Fentanyl 0,2 mg, Midazolam 10 mg und Rocuronium 70 mg.

Hinsichtlich der Vorerkrankungen konnte nach Sichtung der vor Ort gefundenen Dauermedikation (Levetiracetam und Olanzapin) lediglich eine Epilepsie und eine Schizophrenie vermutet werden. Im initialen EKG zeigten sich typische ST-Strecken-Hebungen in mehreren Versorgungsgebieten. Auf die präklinische Antikoagulation mittels Heparin sowie eine Thrombozytenaggregationshemmung wurde bewusst verzichtet, da die notärztliche Kollegin aufgrund der EKG-Veränderungen, einhergehend mit schwerer neurologischer Beeinträchtigung, ein akutes Aortensyndrom vermutete.

**Abb. 1:**
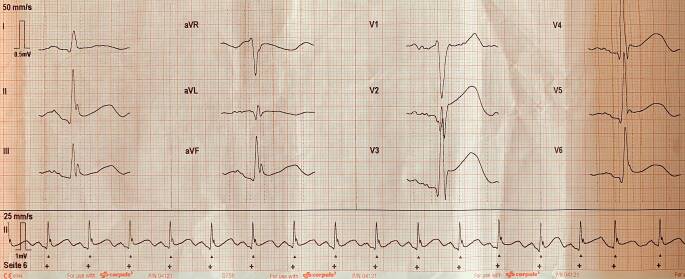
Präklinisches 12-Kanal EKGAbb

Uns wurde eine intubierte und relaxierte Patientin mit stabilen Herz-Kreislauf-Verhältnissen und guter peripherer Oxygenierung übergeben. Im EKG zeigten sich wie bereits angedeutet typische ST-Strecken-Hebungen in V1–V6 und II/III/aVF ohne Dynamik im Verlauf (Abb. 1).

**Abb. 2:**
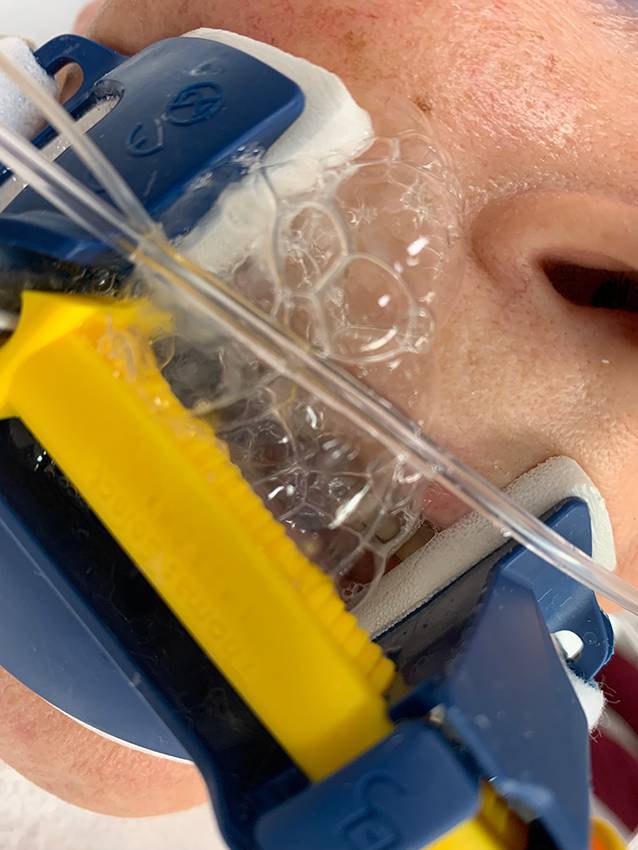
Schaumiges Bronchialsekret im Sinne eines Lungenödems

Die arterielle Blutgasanalyse zeigte neben einer guten Oxygenierung und Ventilation normale Hämoglobin- und Laktatwerte. Die Point-of-Care-Sonographie ergab das Bild einer höchstgradig reduzierten linksventrikulären Ejektionsfraktion (technisch nicht quantifiziert, jedoch visuell ca. 10–15 %) mit septaler, inferiorer und lateraler Hypo- bis Akinesie bei mäßig erhaltener Kontraktilität der Herzspitze. Ein Perikarderguss konnte ausgeschlossen werden. Es zeigte sich keine Aorteninsuffizienz, Aorta ascendens und der Aortenbogen waren von transjugulär angelotet nicht erweitert. Ebenso wenig kam eine Dissektionsmembran zur Darstellung. Pulmonal fand sich regelrechtes Pleuragleiten beidseits bei deutlich vermehrten B‑Linien in den posterobasalen Lungenanteilen. Aus dem Endotrachealtubus konnte schaumiges, klares Sekret abgesaugt werden (Abb. 2).

Die Patientin erhielt unverzüglich eine Kontrastmittel-CT des Schädels sowie der extrakraniellen Halsgefäße und der gesamten Aorta bis zur Bifurkation.

## Wie lautet Ihre Diagnose?

## Therapie und Verlauf

**Abb. 3:**
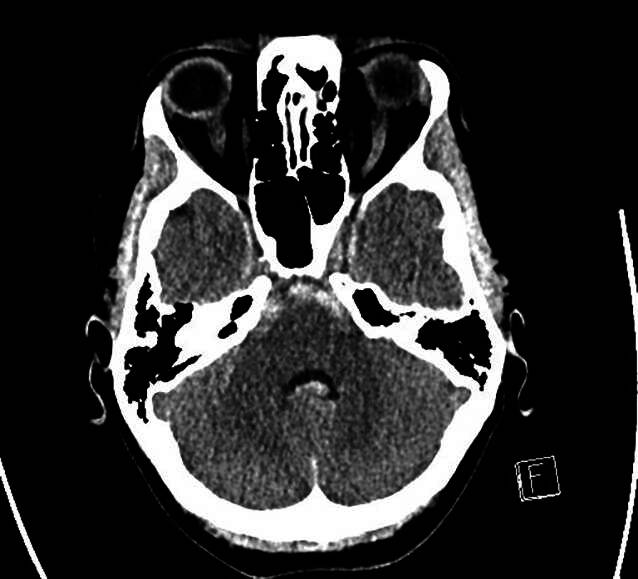
Subarachnoidalblutung ventral des Hirnstamms

Unsere initiale Verdachtsdiagnose einer intrazerebralen Blutung, genauer gesagt eines 5 × 3 mm messenden rupturierten Aneurysmas der linken PICA mit Einblutung ventral des Hirnstamms, konnte CT-morphologisch bestätigt werden (Abb. 3 und 4).

**Abb. 4:**
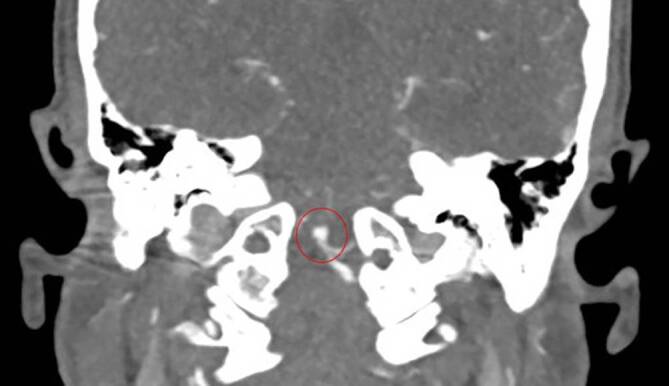
Basilariskopfaneurysma

Es fanden sich bereits Zeichen des Liquoraufstaus. Aufgrund von flau sedimentiertem Blut in den Hinterhörnern der Seitenventrikel war von einer zweizeitigen Subarachnoidalblutung auszugehen.

In Zusammenschau gingen wir aufgrund der diffusen ST-Strecken-Hebungen (mindestens zwei Versorgungsgebiete), nach Ausschluss einer Aortendissektion mit Koronarbeteiligung sowie der höchstgradig reduzierten linksventrikulären Ejektionsfraktion, von einer schweren Takotsubo-Kardiomyopathie aus. Zudem zeigte sich trotz mittlerweile normotensiver Kreislaufverhältnisse das Bild eines neurogenen Lungenödems. Therapeutisch kommt hierfür nur die Hirndrucksenkung durch entsprechende neurochirurgische bzw. neuroradiologische Interventionen infrage. Nach interdisziplinärer Fallbesprechung erfolgte primär die operative Anlage einer rechtsseitigen Ventrikeldrainage und im Anschluss die Versorgung des Aneurysmas mittels Coiling durch die interventionelle Radiologie. Trotz gut nachvollziehbarer Kausalität im Sinne einer schwer geschädigten „neurokardiogenen“ Achse wurde vonseiten der Kardiologie eine Herzkatheteruntersuchung durchgeführt, in welcher eine stenosierende koronare Herzerkrankung ausgeschlossen werden konnte. Die Prognose der Patientin ist in Anbetracht der Blutungslokalisation sowie der initialen Auffindesituation als eher schlecht einzuschätzen, der Heilungsverlauf ist zum jetzigen Zeitpunkt jedoch noch nicht abgeschlossen.

## Differenzialdiagnostik

Die wichtigste initiale Differenzialdiagnose ist in diesem Fall aufgrund der EKG-Veränderungen sowie der deutlich eingeschränkten Herzleistung mit Sicherheit ein akuter Koronarverschluss bzw. ein in die Koronararterien dissezierendes Aortensyndrom. Bei genauerem Hinsehen fällt jedoch auf, dass sich die ST-Strecken-Hebungen sowohl im Versorgungsgebiet der rechten Koronararterie (II/II/aVF) als auch über die gesamte Vorderwand in V1–V6 erstrecken. Ein akutes Aortensyndrom mit Beteiligung beider Koronararterien wäre nicht mit dem Leben vereinbar und widerspricht der initialen Auffindesituation mit eher hypertensiven Blutdruckwerten. Echokardiographisch zeigt sich zwar das Bild einer höchstgradig reduzierten linksventrikulären Ejektionsfraktion; betrachtet man jedoch die segmentalen Abschnitte des linken Ventrikels separat, so sieht man, dass sich deren Kontraktionsverhalten nicht mit dem EKG-Bild deckt. So war beispielsweise lediglich die Kontraktilität der Herzspitze weitestgehend erhalten, was eher untypisch für einen großen „Vorderwandinfarkt“ wäre. Zusammenfassend muss hierbei von einer Takotsubo-Kardiomyopathie bei schwerer intrazerebraler Blutung ausgegangen werden. Präklinisch wurde, wie bereits erwähnt, bewusst auf die Gabe gerinnungshemmender Substanzen verzichtet, obwohl sich elektrokardiographisch eindeutige Zeichen einer schweren Myokardischämie zeigten. Da jedoch die Gesamtsituation völlig untypisch für einen „banalen“ Myokardinfarkt war, sollte das EKG, insbesondere im präklinischen Setting, immer im Kontext (Anamnese, klinische Untersuchung usw.) gesehen werden. Eine unkritische Gabe potenziell schädlicher Medikamente sollte bei hoher Wahrscheinlichkeit für andere, wenn auch seltene Differenzialdiagnosen unterlassen werden.

Klinisch (schaumiger Auswurf) und sonographisch (pathologisch vermehrte B‑Lines) zeigte sich – wie bereits beschrieben – das Bild eines akuten Lungenödems. Differenzialdiagnostisch kommt hierfür neben einer neurogenen Genese selbstverständlich auch ein akutes Linksherz- bzw. Rückwärtsversagen in Betracht. Da sich jedoch auch unter deutlich reduzierter Nachlast sowie im Verlauf etablierter forcierter diuretischer Therapie weiterhin eine deutliche Überwässerung des pulmonalen Interstitiums zeigte, ist unserer Ansicht nach eher von einem neurogenen Lungenödem auszugehen. Eine eindeutige Unterscheidung ist in der Akutsituation in der Notaufnahme weder möglich noch therapeutisch relevant.

## Komplikationen

In der Literatur sind drei Typen der Takotsubo-Kardiomyopathie beschrieben. Am häufigsten findet sich der klassische Typ (66,7 %) mit dem typischen Kontraktionsmuster des „apical ballooning“, das heißt erhaltener basaler Kontraktilität und apikaler Akinesie. In absteigender Reihenfolge finden sich die „Reverse*-*“ bzw. „Inverted-“ (23,3 %) und die „Mid-Cavitary-Takotsubo-Kardiomyopathie“ (10 %), bei welcher die Kontraktilität der mittventrikulären Abschnitte erhalten ist. In unserem Fall liegt vermutlich das Bild einer atypischen bzw. „Reverse*-*Takotsubo-Kardiomyopathie“ vor [1].

Es zeigt sich das spiegelbildliche Kontraktionsverhalten im Vergleich zur typischen Präsentation im Sinne von apikal erhaltener Kontraktilität und basaler Akinesie. Diese Patienten sind häufig postmenopausale Frauen und in der Regel deutlich jünger, verglichen mit Patienten mit anderen Formen (54,5 versus 64 Jahre). Sie weisen laut Literatur nahezu immer starke mentale oder physische Stressoren als Trigger auf [2, 3].

Erschwerend zur Grunderkrankung zeigte sich bereits bei Auffindesituation das Bild eines akuten Lungenödems mit schwerer respiratorischer Partialinsuffizienz und schaumigem Auswurf. Dieses hatte auch unter kontrollierter Beatmung mit einem PEEP von 5 cmH_2_O und unter normotensiven Kreislaufverhältnissen während des Transports bis zur Ankunft in unserer Notaufnahme Bestand.

Das neurogene Lungenödem ist definiert als seltene Form der akuten respiratorischen Insuffizienz, welche aufgrund einer massiven sympathikotonen Überstimulation mit ausgeprägter systemischer und vor allem pulmonalarterieller Vasokonstriktion durch akute Schädigung des zentralen Nervensystems ausgelöst wird. Durch die massive Nachlasterhöhung kommt aus zu einer Zunahme des linksatrialen Drucks, welcher sich in Form eines erhöhten Verschlussdrucks (Wedge-Druck) in den kleinen Kreislauf fortsetzt. Durch den damit erhöhten pulmonalvaskulären Widerstand sowie lokaler Ausschüttung von neurohumoralen Substanzen (u. a. Histamin und Bradykinin) kommt es schließlich zu einem ausgeprägten „capillary leak“ mit Flüssigkeits-Shift ins Interstitium.

Die kausale Therapie ist die Behandlung der Grunderkrankung bzw. die zügige Hirndrucksenkung. Die Prognose ist aufgrund der Schwere der zugrunde liegenden Erkrankung schlecht [4].

**Diagnose:** Rupturiertes Basilariskopfaneurysma mit neurogenem Lungenödem und atypischer Takotsubo-Kardiomyopathie

Die Inzidenz eines neurogenen Lungenödems bei intrazerebraler Blutung ist in der Literatur mit 8 % angegeben und war signifikant höher bei Patienten mit intrazerebraler Blutung im posterioren Stromgebiet. Das neurologische Outcome ist signifikant schlechter als bei Patienten ohne neurogenes Lungenödem [5].

## Fazit für die Praxis

- „STEMI mimics“ sollten insbesondere bei untypischer Gesamtkonstellation an weitere lebensbedrohliche Differenzialdiagnosen denken lassen.
- Akute intrakranielle Pathologien bedingen in relevanter Zahl auch akute Organschäden andernorts (Herz, Lunge), welche die Patientenversorgung zusätzlich verkomplizieren können.
- Die bettseitige Notfallsonographie ist bei jedem kritisch kranken Patienten anzuwenden, um die Zahl an bedrohlichen Differenzialdiagnosen eingrenzen zu können.
